# Differential Physiological, Transcriptomic, and Metabolomic Responses of *Paspalum wettsteinii* Under High-Temperature Stress

**DOI:** 10.3389/fpls.2022.865608

**Published:** 2022-04-21

**Authors:** Xin Zhao, Li-Juan Huang, Xiao-Fu Sun, Li-Li Zhao, Pu-Chang Wang

**Affiliations:** ^1^College of Animal Science, Guizhou University, Guiyang, China; ^2^School of Life Sciences, Guizhou Normal University, Guiyang, China

**Keywords:** *Paspalum wettsteinii*, high-temperature stress, physio-biochemical, transcriptomic, metabolomic

## Abstract

Global warming has far-reaching effects on plant growth and development. As a warm-season forage grass, *Paspalum wettsteinii* is highly adaptable to high temperatures. However, the response mechanism of *P. wettsteinii* under high-temperature stress is still unclear. Therefore, we investigated the physiological indicators, transcriptome and metabolome of *P. wettsteinii* under different heat stress treatments. Plant height, the activities of superoxide dismutase (SOD), peroxidase (POD), and catalase (CAT), and the contents of soluble sugar, proline, chlorophyll *a*, and chlorophyll *b* increased and then decreased, while the malondialdehyde (MDA) content decreased and then increased with increasing heat stress. Transcriptomic analysis revealed that genes related to energy and carbohydrate metabolism, heat shock proteins (HSPs), and transcription factors (TFs), secondary metabolite biosynthesis and the antioxidant system significantly changed to varying degrees. Metabolomic analysis showed that only free fatty acids were downregulated, while amino acids and their derivatives, organic acids, flavonoids, and sugars were both up- and downregulated under heat stress. These combined analyses revealed that growth was promoted at 25–40°C, while at 45°C, excess reactive oxygen species (ROS) damage reduced antioxidant and osmoregulatory effects and inactivated genes associated with the light and electron transport chains (ETCs), as well as damaged the PS II system and inhibited photosynthesis. A small number of genes and metabolites were upregulated to maintain the basic growth of *P. wettsteinii*. The physiological and biochemical changes in response to high-temperature stress were revealed, and the important metabolites and key genes involved in the response to high temperature were identified, providing an important reference for the physiological and molecular regulation of high-temperature stress in plants.

## Introduction

The growth and development of plants are susceptible to external environmental stresses such as biotic stresses as well as abiotic stresses caused by extreme temperatures, drought, and heavy metals (Kim et al., 2021). Among the many adverse factors, heat stress is one of the most severe and least controllable factors affecting plant growth and reproduction (Herman et al., 2017). Climate change has increased the frequency of extreme heat across the globe (Easterling et al., 2000), exacerbating its damage and limiting plant growth, metabolism, quality, and yield (Zhao et al., 2016). The adaptation of plants to high-temperature stress and their tolerance mechanisms have become a research hotspot in many fields, such as the environment, ecology, and genetics, under the increasingly severe global heat threat (Chen et al., 2022).

Plants, as sedentary organisms, have evolved complex and diverse systems to respond to high temperatures (Aranguren-Gassis et al., 2019). For example, many plants mature relatively early under high-temperature conditions to avoid the effects of heat, and most plants exhibit similar changes under heat stress, such as reduced cell size, tighter arrangement, closure of stomata, reduced photosynthesis and more developed xylem conduits in roots and stems (Bañon et al., 2004). At the same time, plants produce a range of antioxidant enzymes and antioxidants to scavenge ROS and protect cells from damage caused by peroxidative reactions; for example, the balance between superoxide dismutase (SOD), peroxidase (POD), or catalase (CAT) is essential for scavenging superoxide radicals and hydrogen peroxide (Almeselmani et al., 2015), by accumulating some osmoregulatory substances (proline, betaine, soluble sugars, etc.) to mitigate osmotic stress injury (Sezgin and Kadioglu, 2021); for example, betaine maintains barley seed cell stability and increases the photosynthetic rate and leaf water potential under high-temperature stress; proline reduces chickpea cell damage and protects some important enzymes related to carbon and oxidative metabolism; and an increase in soluble sugars improves the heat tolerance of sugarcane (Wahid and Close, 2007; Meng et al., 2012).

In addition, at the molecular level, plants integrate stress signals to activate the expression of a range of stress-responsive genes and other cellular activities through transcriptomic and metabolomic pathways, protecting plant cells by re-establishing intracellular homeostasis and repairing cellular components (Wan et al., 2020). Wang et al. (2020) found that differentially expressed transcription factors (TFs), including the bZIP, WRKY, DREB, and HSF gene families and HSPs, were differentially expressed in *Arabidopsis* leaves in response to high-temperature stress. Hu et al. (2020) found that the differential expression analysis of tall fescue genes identified 19 key genes for heat tolerance, including PFK, ALDO, PGLS, gltA, ACSL, and others. In addition, Liao et al. (2015) used microarray technology to functionally annotate differentially expressed genes (DEGs) in rice flag leaves under different high-temperature stresses and found that genes related to the glycolytic pathway, heat kinase, and ubiquitin proteasome were upregulated, while genes involved in carotenoid, dihydroflavonol, anthocyanin metabolism, photosynthetic light response, and TCA cycle were downregulated. In addition, the transcriptomes of cotton (Ma et al., 2021), *Ananas comosus* (Zhao et al., 2021), *Pyropia haitanensis* (Chang et al., 2021), and *Brassica napus* (Gao et al., 2021) have also been analyzed in depth, and key pathways and their response genes have been identified.

Volatiles released by plants through secondary metabolism are also important for plant resistance to adverse conditions, and the accumulation of metabolites in plants under high-temperature stress conditions changes significantly. Many metabolites, such as organic acids, amino acids, sugars, sugar alcohols, polyamines, and lipids, are involved in the plant stress response (Du et al., 2011; Gao et al., 2021). Chebrolu et al. (2016) studied soybean seed development metabolites under heat stress and found that a variety of antioxidant metabolites, such as flavonoids and phenylpropanoids, were monitored in heat-tolerant soybeans. Escandón et al. (2018) used complementary mass spectrometry (GC–MS and LC-Orbitrap-MS) to metabolomically sequence *Pinus radiata* treated at 40°C and found that cytokinins (CKs), fatty acid metabolism, and flavonoid and terpene biosynthesis were key pathways in the heat stress response and successfully identified heat-tolerant metabolites such as hexadecanoic acid and dihydromyricetin. In addition, key metabolic pathways and metabolites have been proposed for high-temperature stress in *Cynodon dactylon* (Du et al., 2011), *Zostera marina* (Gao et al., 2019), and *Pinus radiata* (Escandón et al., 2018).

*Paspalum wettsteinii* is a warm-season grass herb of the genus *Paspalum* with strong tolerance to salinity, infertility, acid and drought, a well-developed root system, fast growth rate, strong tiller and regeneration and is a pioneer plant for ecological vegetation restoration. Second, *P. wettsteinii* is fast growing, has good palatability, is rich in nutrients, has high grass production, has a high C/N content, is good for organic matter accumulation, has a high humification coefficient, has a fertilizing effect on the soil, can promote the formation of good soil structure and is a high-quality forage grass and green manure that is promoted and used in southern China (Zhao et al., 2019; Sun et al., 2020). At present, studies of high-temperature stress on *P. wettsteinii* have mainly focused on morphological structure and physiological and biochemical responses (Sun et al., 2020), but the molecular mechanisms are still unclear. Based on these facts, we investigated the transcriptional regulation and metabolic changes under high-temperature stress and elucidated the regulatory mechanism of the response of *P. wettsteinii* to high-temperature stress. In this experiment, we used *P. wettsteinii* as a research target and conducted high-temperature treatment on potted seedlings. Through transcriptomic and metabolomic analyses, we revealed the presence of core heat resistance candidate genes and key metabolites in young leaves of *P. wettsteinii* under high-temperature stress, which provides an important reference for the study of high-temperature stress in warm-season plants.

## Materials and Methods

### Plant Materials and Experimental Design

*wettsteinii* seeds were obtained from the Grassland Science Laboratory of the College of Animal Science of Guizhou University of China and planted in plastic pots (49 cm in length × 20 cm in width × 13 cm in height) at Guizhou University (26°27′N, 106°39′E), Huaxi district, Guiyang, Guizhou Province, China. *Paspalum wettsteinii* plants were grown in a mixed culture of humus, vermiculite and natural soil (1:1:1, v:v:v) in a greenhouse at 25/20°C (day/night) under natural sunlight and a relative humidity of 70%. After growth to two leaves, one heart was transferred into controlled-environment growth chambers (BIC-400, Boxun Instrument, Shanghai, China) at 75% humidity and 25°C/20°C (day and night) under a light:dark cycle of 12 h:12 h and an illumination of 30000 Lux at the canopy level to acclimate for 1 week before heat treatments were imposed. The soil was watered with 1/2 Hoagland solution once a week during the incubation process. All soils were fully irrigated with water once the day before the heat treatment, and the pots and seedlings were weighed separately after being left overnight to determine the soil water content. The soil water content during heat treatment was maintained daily by weighing and rehydrating the soil during heat treatment. All *P. wettsteinii* plants were divided into four groups. The control (CK, 25/20°C) kept the environment unchanged. LH, MH, and SH plants were subjected to heat treatment at 35/25°C, 40/35°C, and 45/40°C (day and night) in controlled-environment growth chambers (except for temperature, the other conditions remained the same as the control). Heat treatment began at 7:00 a.m. After 4 days, the upper leaves were harvested in seedlings (four control and treatment) with material homogeneity. The leaf samples were immediately frozen in liquid nitrogen and stored at −80°C. There were three biological replicates per treatment for physiological RNA-seq analysis and LC–MS analysis. 7G data were used for Illumina Genon analyzer deep sequencing of each sample.

### Plant Height Determination

Plant height (the vertical height from the ground to the top of the plant) was measured with a ruler. In our study, plant height refers to the average daily growth height during the 4 days of high-temperature stress.

### Measurements of Physio-Biochemical Characteristics

The activities of antioxidant enzymes, including SOD (EC 1.15.1.1), POD (EC 1.11.1.7), and CAT (EC 1.11.1.6), were determined using the superoxide dismutase, peroxidase, and catalase activities detection kit’s instructions (Beijing Solarbio Science & Technology Co., Ltd.). Fresh leaves (0.1 g) were extensively homogenized in 50 mM phosphate-buffered saline (PBS), which were ground into a slurry on ice and centrifuged at 8,000 × *g* at 4°C for 10 min. The supernatant was collected, aliquoted, and then stored in an ultralow-temperature refrigerator for activity analysis. SOD activity was determined by a mixture of 50 mM PBS, enzyme solution, and riboflavin solution. The absorbance was measured at 560 nm. The reaction system for the POD activity assay consisted of 100 mM PBS (pH = 6.0), 20 mM guaiacol and 40 mM H_2_O_2_. During the assay, the reaction solution was taken and preheated in a water bath at 25°C for 5 min, and then, the enzyme solution was added to initiate the reaction. The change in absorbance per unit time was measured at 470 nm. For the CAT activity assay, the reaction mixture (50 mM PBS containing 0.1 mM EDTA, pH = 7.8) was preheated in a water bath at 25°C for 5 min, and then, the enzyme solution was added. The reaction was initiated with 19 mM H_2_O_2_, and the change in absorbance per unit time at 240 nm was measured.

Osmotic adjustment substances including proline and soluble sugar. Proline was determined using the proline content detection kit’s instructions (Beijing Solarbio Science & Technology Co., Ltd.). Fresh leaves (0.1 g) were homogenized in 3% sulfosalicylic acid. The filtrate was reacted in response to 1 ml and acid ninhydrin. After extraction with toluene, the absorbance was measured at 520 nm. Proline contents were determined by comparing the absorbance value with the calibration plot for standard solutions. The soluble sugar content was determined using the soluble sugar content detection kit’s instructions (Beijing Solarbio Science & Technology Co., Ltd.). Fresh leaves (0.1 g) were homogenized in 1 ml distilled water. Boiling water bath for 10 min, cooled, 8,000 *g*, centrifuged at room temperature for 10 min, the supernatant was taken in a 10 ml test tube, fixed with distilled water, and the absorbance was measured at 620 nm using the above kit.

The chlorophyll (chlorophyll *a* and chlorophyll *b*) content was determined using the chlorophyll content detection kit’s instructions (Beijing Solarbio Science & Technology Co., Ltd.). Fresh leaves were extensively homogenized in 80% acetone and leached for 3 h in the dark. The homogenate was centrifuged in the dark at 1,500 × *g* for 5 min, and the OD of the supernatant was measured at 645 and 663 nm.

The malondialdehyde (MDA) content was determined using the malondialdehyde content detection kit’s instructions (Beijing Solarbio Science & Technology Co., Ltd.). Fresh leaves (0.1 g) were homogenized in trichloroacetic acid (TCA; 0.1%). Then, 0.5% TBA was added to the supernatant. The mixture was heated, cooled, and then centrifuged at 10,000 × *g* for 5 min. The absorbance was read at 532 nm.

### RNA Extraction, RNA-Seq, and Bioinformatics Analysis

Four leaf samples from each high-temperature treatment were used for RNA-seq. Total RNA was extracted from each sample using TRIzol reagent (Invitrogen, United States). After RNA quality assessment, mRNA purification, and mRNA fragmentation, a cDNA library was constructed, and raw data were obtained by Illumina paired-end sequencing on an Illumina HiSeq 4000 platform. Clean reads were obtained by removing reads containing adapters and reads containing poly-N sequence reads of low quality from the raw data. Gene expression levels in each sample were estimated by RSEM (v1.2.15). The clean data were mapped back into the assembled transcriptome using RSEM. The read counts for each sequence were then obtained from the mapping results by Bowtie2 (with a mismatch of 0) and normalized to reads per kilobase of exon model per million mapped reads (RPKM). Differential expression analysis of the two conditions/groups was performed using the DESeq R package (1.10.1). The resulting *p* values were adjusted using Benjamini and Hochberg’s approach to control the false discovery rate (FDR). The value of *p* was adjusted using the *Q*-value, with value of *Q* < 0.005 and |log2 > 1| set as the thresholds for significant differences in gene expression. Correlation analysis and principal component analysis (PCA) were used to test the reliability of the samples. DEGs were identified in the LH/CK, MH/CK, and SH/CK groups. For functional analysis, all the DEGs were subjected to Gene Ontology (GO) annotation^1^ and Kyoto Encyclopedia of Genes and Genomes (KEGG)^2^ pathway enrichment analyses. The GO and KEGG pathway enrichment results were considered significant when *p*-values adjusted *via* Bonferroni correction (*Q*-values) were ≤0.05, and WEGO software was used for statistical analysis of the GO functional categories and KEGG pathway enrichment results.

### Quantitative RT–PCR Analysis

Eighteen DEGs were selected to validate the RNA-seq results (Supplementary Table S1). cDNA was synthesized from the same RNA samples used for transcriptome sequencing. We performed quantitative real-time PCR (qRT–PCR) on a CFX Connect™ Real-Time System (Applied Biosystems) using UltraSYBR mixture (CWBiotech). The thermocycle parameters were 10 min at 95°C, 40 cycles of 10 s at 95°C, 30 s at 60°C, and 32 s at 72°C, followed by 15 s at 95°C, 1 min at 60°C, 15 s at 95°C, and 15 s at 60°C, in a 20 μl reaction mixture. The *Arabidopsis thaliana*-actin gene was selected as an internal standard for normalization.

### Metabolite Identification and Quantification by LC–MS

Widely targeted metabolomic profiling was carried out by a commercial service company (Biomarker Technologies, Beijing, China). Briefly, 100 mg of leaves {24 samples [four treatments (CK, LH, MH, and SH)] × six biological replicates} was individually ground in liquid nitrogen, and the homogenates were resuspended in prechilled 80% methanol and 0.1% formic acid thorough vortexing. The samples were incubated on ice for 5 min and then centrifuged at 15,000 × *g* at 4°C for 20 min. A portion of the supernatant was diluted with LC–MS-grade water such that the final constitution was equal to 53% methanol. The samples were subsequently transferred to a new Eppendorf tube and then centrifuged again at 15,000 × *g* at 4°C for 20 min. Finally, the supernatant was injected into the HPLC–MS/MS system, and analysis was performed using a Vanquish UHPLC system (Thermo Fisher, Germany) equipped with an Orbitrap Q ExactiveTM HF-X mass spectrometer (Thermo Fisher, Germany). The HPLC and MS conditions were the same as those used by Yamada and Nishimura (2008). Compound Discoverer 3.1 (CD 3.1; Thermo Fisher) was used for peak alignment, peak picking and quantitation of each metabolite. The metabolites were subsequently annotated using the KEGG database.^3^ PCA and partial least squares discriminant analysis (PLS-DA) were performed with metaX (dynamic and comprehensive software for processing metabolomic data). We applied univariate analysis (*t*-test) to calculate the statistical significance (*p*-value). Metabolites with a VIP > 1, a value of *p* < 0.05 and a fold-change (FC) ≥ 2 or ≤0.5 were considered to be differentially accumulated.

### Statistical Analysis

The data were collated using WPS office software and SPSS 25.0 software for one-way ANOVA statistical analysis and the least significant difference (LSD) method for multiple comparisons, with significant differences defined at *p* < 0.05. All data are the means ± SEs of three replicates, and SigmaPlot 14.0 was used for graphical plotting.

## Results

### Physiological and Biochemical Changes

**Figure 1:**
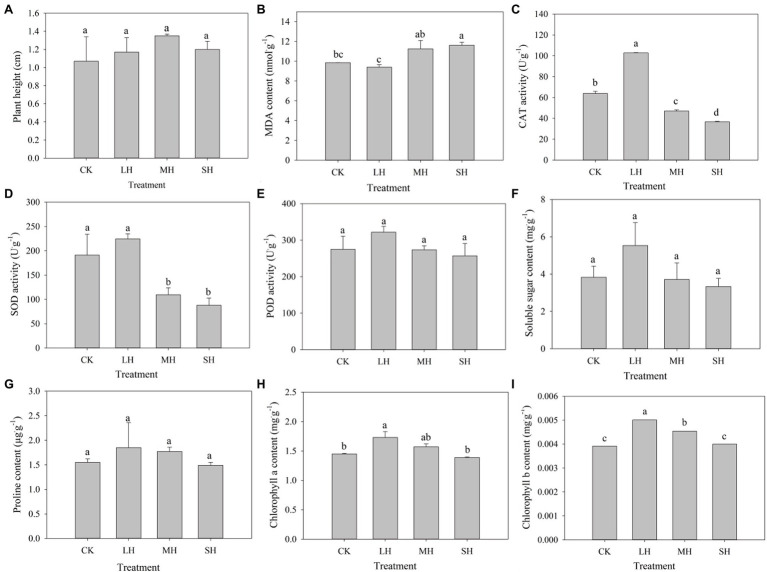
Plant height and physio-biochemical characteristic changes in *Paspalum wettsteinii* under high-temperature stress. Values are means ± SEs (*n* = 3). The different lowercase letters for each treatment are significant at least significant difference (LSD) test at *p* < 0.05. Effects of drought stress on **(A)** plant height, **(B)** MDA content, **(C)** CAT activity, **(D)** SOD activity, **(E)** POD activity, **(F)** soluble sugar content, **(G)** proline content, **(H)** chlorophyll a content, and **(I)** chlorophyll b content.

To measure the effect of heat stress on the *P. wettsteinii* growth rate, plant height was examined before and after 4 days of treatment to calculate the growth rate. We found that the growth rate of plant height increased and then decreased with increasing heat stress, reaching a maximum in the MH treatment, but the difference between all four treatments was not significant (Figure 1A).

The MDA content tended to decrease and then increase with increasing heat stress. Compared to that in the CK group, the MDA content decreased with LH treatment, but the difference was not significant, while it increased with MH and SH treatments, but only the difference was significant with SH treatment (Figure 1B). The activities of CAT, SOD, and POD and the contents of soluble sugar, proline, chlorophyll *a*, and chlorophyll *b* tended to increase and then decrease with increasing heat stress, and all reached a maximum in the LH treatment. Compared to those in the CK group, the activities of CAT, SOD, and POD and the soluble sugar content increased with LH treatment and decreased with MH and SH treatments. CAT activity was significantly different in all three stress treatments, and SOD was significantly different in the MH and SH treatments (Figures 1C–F). Compared to those in the CK group, the SP and chlorophyll *a* contents increased with LH and MH treatment and decreased with SH treatment, and the chlorophyll *a* content was significantly different with LH treatment (Figures 1G,H). Compared to those in the CK group, the chlorophyll b content significantly increased in the LH and MH treatments (Figure 1I).

### DEGs Identified in *Paspalum wettsteinii*

To identify genes responding to high-temperature stresses, 12 cDNA libraries were generated with mRNA from four samples of CK, LH, MH, and SH treatments. The same materials used in the transcriptomic analysis were sequenced, yielding a total of 286 million high-quality clean reads from three replicates of 12 libraries. The sequencing data were deposited in NCBI (SRA accession: PRJNA801753), and the Q30 values of the samples reached 92%. Approximately 67% of the reads from each library were perfectly matched to the reference genome (Supplementary Table S2). A total of 18 DEGs were randomly selected for qRT–PCR validation (Supplementary Table S1; Supplementary Figure S1). Their expression patterns were highly correlated with the fragments per kilobase per million reads (FPKM) values of the RNA-seq data, corroborating the reliability of the transcriptomic data.

A total of 3,653 DEGs (1,791 upregulated and 1,862 downregulated) were identified in *P. wettsteinii* (Supplementary Figure S2). For LH/CK, 334 DEGs, including 140 genes that were obviously upregulated and 194 genes that were obviously downregulated, were detected (Supplementary Figure S2A). For MH/CK, 847 DEGs were differentially expressed (448 upregulated and 399 downregulated; Supplementary Figure S2B). For SH/CK, 2472 DEGs were differentially expressed (1,203 upregulated and 1,269 downregulated; Supplementary Figure S2C). Among these DEGs, only 168 common genes were differentially expressed among all three treatment groups (Supplementary Figure S2A; Supplementary Table S3). These common DEGs were considered valuable candidate genes for improving high-temperature tolerance.

Gene Ontology is widely used in gene functional annotation and enrichment analysis. The main role of GO was to classify the functions of the predicted *P. wettsteinii* genes. The identified GO terms were associated with three major categories: biological processes, cellular components, and molecular functions. In total, 184 of the 334 DEGs in the LH/CK group, 527 of the 847 DEGs in the MH/CK group and 1,445 of the 2,472 DEGs in the SH/CK group were assigned to at least one GO term during high-temperature stress. In the LH/CK group, all significantly enriched DEGs were assigned to cellular components, biological processes, and molecular functions, and 11, 16, and 10 GO terms were identified in these groups, among which cell, cell part, catalytic activity, and binding were the four most enriched subcategories (Supplementary Table S4). In the MH/CK group, all significantly enriched DEGs were assigned to cellular components, biological processes, and molecular functions, and 14, 18, and 12 GO terms were identified, respectively, among which cell, cell part, catalytic activity, and metabolic process were the four most enriched subcategories (Supplementary Table S4). In the SH/CK group, all significantly enriched DEGs were assigned to cellular components, biological processes, and molecular functions, and 16, 18, and 13 GO terms were identified, respectively, among which cell, cell part, catalytic activity, and metabolic process were the four most enriched subcategories (Supplementary Table S4). The same enriched GO terms were associated with different functional categories in these three treatment groups, suggesting that these GO terms were involved in the response and tolerance to high-temperature stress and play a vital role in the *P. wettsteinii* response to different degrees of high-temperature stress.

Kyoto Encyclopedia of Genes and Genomes pathway enrichment analysis is an effective method for elucidating the biological functions of DEGs. Therefore, the DEGs enriched in KEGG pathways according to various biological functions were further analyzed. DEGs with a value of *p* ≤ 0.05 were defined as significantly differentially expressed. In our study, among the KEGG enrichment pathways of the LH/CK, MH/CK, and SH/CK comparison groups, 8, 17, and 19 significantly enriched pathways (*p* < 0.05), respectively, were identified (Supplementary Table S5). As shown in Supplementary Figures S3A-C, we screened the top 20 enriched KEGG pathways for the LD/CK, MD/CK, and SD/CK comparison groups. In the LH/CK comparison group, there were three significantly enriched pathways with a large number of DEGs: linoleic acid metabolism (ko00591, three genes), alpha-linolenic acid metabolism (ko00592, four genes), and flavonoid biosynthesis (ko00941, two genes; Supplementary Figure S3A). In the MH/CK comparison group, phenylpropanoid biosynthesis (ko00940, eight genes), cysteine and methionine metabolism (ko00270, six genes), flavonoid biosynthesis (ko00941, five genes), phenylalanine metabolism (ko00360, five genes), and glycerolipid metabolism (ko00561, five genes) were the five main pathways (Supplementary Figure S3B). In the SH/CK comparison group, phenylpropanoid biosynthesis (ko00940, 21 genes), biosynthesis of amino acids (ko01230, 29 genes), starch and sucrose metabolism (ko00500, 18 genes), ribosome biogenesis in eukaryotes (ko03008, 15 genes), and phenylalanine metabolism (ko00360, 10 genes) were the five main pathways (Supplementary Figure S3C). The KEGG pathway enrichment results thus showed that genes in *P. wettsteinii* were significantly differentially expressed in the LH/CK, MH/CK, and SH/CK comparison groups, indicating that the three groups were activated by different molecular mechanisms under high-temperature stress. In this experiment, it was speculated that these pathways might play an important role in the response of *P. wettsteinii* plants to high-temperature stress.

### Analysis of DEGs Associated With Energy Metabolism

**Figure 2:**
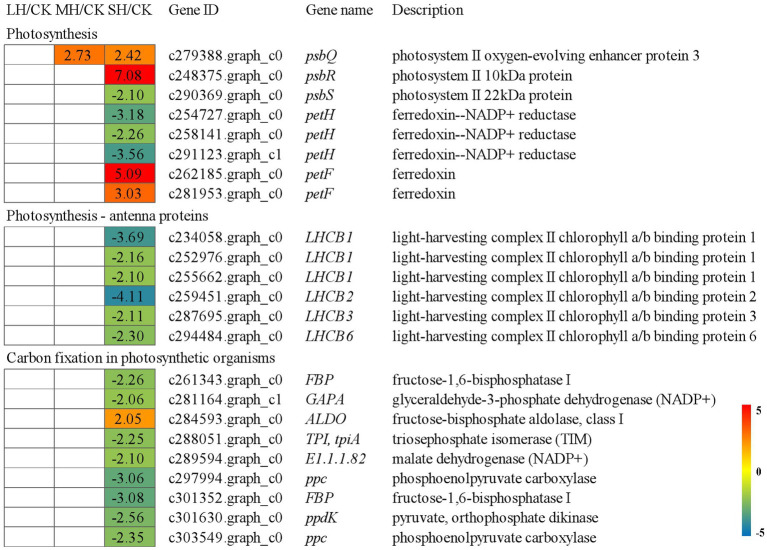
Energy metabolism-related genes identified in the RNA-seq that showed expression changes during temperature treatments. The numbers in the rectangles indicate the log_2_(FC) of the comparisons.

Regarding the “photosynthesis” group, PS II genes, including *psbQ*, *psbR*, and *psbS*, were studied, and *psbQ* was significantly upregulated in both the MH/CK and SH/CK comparison groups, *psbR* was significantly upregulated in the SH/CK comparison group and *psbS* was significantly downregulated in the SH/CK comparison group. The expression levels of other genes involved in photosynthetic electron transport, such as *PetF* and *PetH*, were also significantly altered in the SH/CK comparison group; the three *PetH*s were significantly downregulated in the SH/CK comparison group, and the two *PetF*s were significantly upregulated in the SH/CK comparison group. Regarding the “photosynthesis-antenna proteins” group, six genes of the light-harvesting chlorophyll protein complex, three *LHCB1*s, *LHCB2*, *LHCB3*, and *LHCB6*, were significantly downregulated in the SH/CK comparison group. A total of nine genes involved in carbon fixation were differentially expressed under the SH/CK comparison group; *FBP*, *GAPA*, *tpiA*, *E1.1.1.82*, *ppc*, *FBP*, and *ppdK* were significantly downregulated, whereas *ALDO* was significantly upregulated in SH/CK (Figure 2).

### Heat Shock-Related Genes and Transcription Factors in Response to High-Temperature Stress

**Figure 3:**
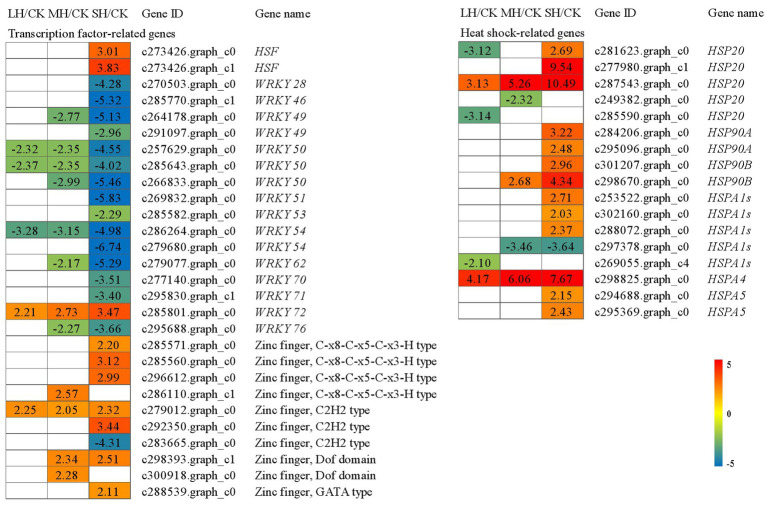
Transcription factor (TF) and heat shock protein (HSP) genes identified in the RNA-seq that showed expression changes during temperature treatments. The numbers in the rectangles indicate the fold-changes (FCs) of the comparisons.

Heat shock proteins (HSPs) are known to contribute to heat resistance. A total of 17 HSP genes were induced under high temperatures. Among the five *HSP20*s, one (c281623.graph_c0) was significantly downregulated in LH/CK but upregulated in SH/CK, one (c277980.graph_c1) was significantly upregulated in SH/CK, one (c287543.graph_c0) was significantly upregulated in the three comparison groups and two (c249382.graph_c0 and c285590.graph_c0) were downregulated in LH/CK or MH/CK. Among the five *HSPA1*s, three (c253522.graph_c0, c302160.graph_c0, and c288072.graph_c0) were significantly upregulated in SH/CK, one (c297378.graph_c0) was significantly downregulated in both MH/CK and SH/CK and one (c269055.graph_c4) was significantly downregulated in LH/CK. Two *HSP90A*s, two *HSP90B*s and two *HSP90A5*s were significantly upregulated in SH/CK. *HSP90A4* was significantly upregulated in the three comparison groups (Figure 3).

In total, 28 transcription factors were identified that belonged to the HSF, WRKY, and zinc finger protein genes. Among HSF, two HSFs were significantly upregulated in SH/CK. Among WRKYs, *WRKY28*, *WRKY46*, *WRKY49* (c291097.graph_c0), *WRKY51*, *WRKY53*, *WRKY54* (c279680.graph_c0), *WRKY70*, and *WRKY71* were significantly downregulated in SH/CK, *WRKY49* (c264178.graph_c0), *WRKY50* (c266833.graph_c0), *WRKY62*, and *WRKY76* were significantly downregulated in MH/CK and SH/CK and two *WRKY50*s and one *WRKY54* were significantly downregulated in the three comparison groups. Among 10 zinc finger protein genes, three C-x8-C-x5-C-x3-H type and one GATA type were significantly upregulated in SH/CK, one Dof domain (c298393.graph_c1) was significantly upregulated in MH/CK and SH/CK, one Dof domain (c300918.graph_c0) was significantly upregulated in MH/CK, one C2H2 type (c279012.graph_c0) was significantly upregulated in three comparison groups and one C2H2 type (c292350.graph_c0) was significantly upregulated in SH/CK, but one C2H2 type (c283665.graph_c0) was significantly downregulated in SH/CK (Figure 3).

### Analysis of DEGs Associated With the Biosynthesis of Secondary Metabolites

**Figure 4:**
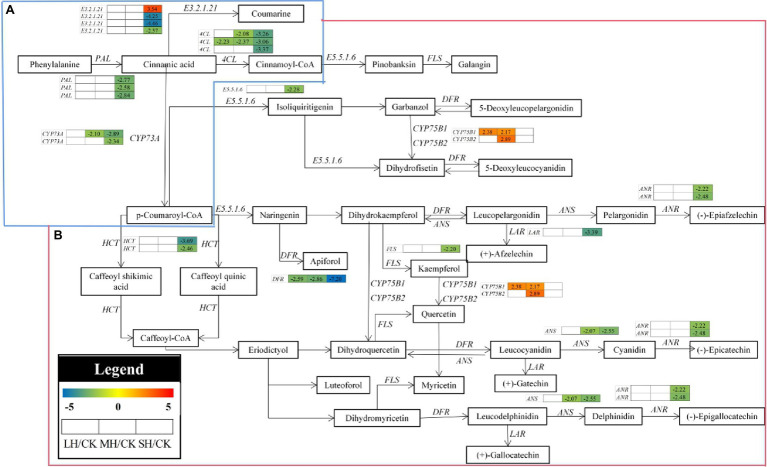
The pathways of phenylpropanoid biosynthesis **(A)** and flavonoid biosynthesis **(B)** enriched by Kyoto Encyclopedia of Genes and Genomes (KEGG) analysis. *CYP75B1*, flavonoid 3′-monooxygenase; *CYP75B2*, flavonoid 4′-monooxygenase; *ANS*, anthocyanidin synthase; *DFR*, bifunctional dihydroflavonol 4reductase/flavanone 4-reductase; *CYP73A*, trans-cinnamate 4-monooxygenase; *HCT*, shikimate O-hydroxycinnamoyltransferase; *LAR*, leucoanthocyanidin reductase; *FLS*, flavonol synthase; *ANR*, anthocyanidin reductase; *E5.5.1.6*, chalcone isomerase; *4CL*, 4-coumarate-CoA ligase; *E3.2.1.21*, beta-glucosidase; and *PAL*, phenylalanine ammonia-lyase. The numbers in the gray rectangles indicate the log_2_(fold-changes) of the comparisons. The genes in the picture are all significantly different in the comparisons.

In the phenylpropanoid and flavonoid biosynthesis pathway, 25 enzyme genes, including *E3.2.1.21*, *PAL*, *4CL*, *CYP73A*, *CYP75B1*, *CYP75B2*, *ANS*, *DFR*, *HCT*, *LAR*, *FLS*, *ANR*, and *E5.5.1.6*, were differentially accumulated in the *P. wettsteinii* leaves under high-temperature stress (Figure 4; Supplementary Table S6). Overall, at the genetic level, phenylpropanoid, and flavonoid metabolism in *P. wettsteinii* leaves were repressed under high-temperature stress since most of the structural genes involved in the pathways were significantly downregulated, whereas *CYP75B1* and *CYP75B2* were significantly upregulated in LH/CK and MH/CK. Interestingly, among *E3.2.1.21*, three *E3.2.1.21*s were significantly downregulated, but one *E3.2.1.21* was significantly upregulated in SH/CK. These results might suggest that phenylpropanoid and flavonoid metabolisms were triggered as defence mechanisms in response to high-temperature stress in *P. wettsteinii*.

### Analysis of DEGs Associated With Carbohydrate Metabolism

**Figure 5:**
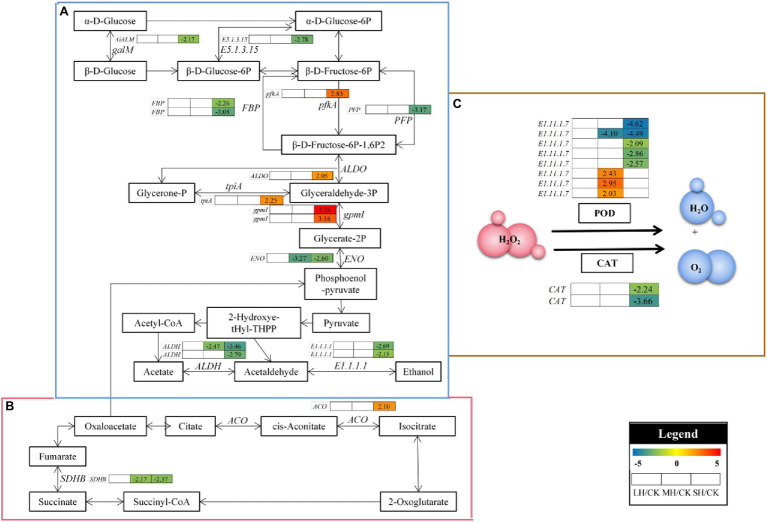
The carbohydrate metabolism and scavenging free radical pathway analysis under high-temperature stress. **(A)** Glycolysis/gluconeogenesis. **(B)** Citrate cycle. **(C)** Profiles of antioxidant enzyme-related genes responsible for scavenging free radicals. *FBP*, fructose-1,6-bisphosphatase I; *gpmI*, 2,3-bisphosphoglycerate-independent phosphoglycerate mutase; *E5.1.3.15*, glucose-6-phosphate 1-epimerase; *ENO*, eno enolase; *ALDO*, fructose-bisphosphate aldolase, class I; *GALM*, aldose 1-epimerase; *E1.1.1.1*, adh alcohol dehydrogenase; *tpiA*, triosephosphate isomerase (TIM); *pfkA*, 6-phosphofructokinase 1; *ALDH*, aldehyde dehydrogenase (NAD+); *PFP*, diphosphate-dependent phosphofructokinase; *SDHB*, succinate dehydrogenase (ubiquinone) iron–sulfur subunit; and *ACO*, acnA, aconitate hydratase. *E1.11.1.7*, peroxidase; and *CAT*, catalase. The numbers in the gray rectangles indicate the log_2_(fold-changes) of the comparisons. The genes in the picture are all significantly different in the comparisons.

In the glycolysis/gluconeogenesis and citrate cycle pathway, 17 enzyme genes, including *FBP*, *gpmI*, *E5.1.3.15*, *ENO*, *ALDO*, *GALM*, *E1.1.1.1*, *tpiA*, *pfkA*, *ALDH*, *PFP*, *SDHB*, and *ACO*, were differentially accumulated in *P. wettsteinii* leaves under high-temperature stress (Figure 5; Supplementary Table S7). Overall, at the genetic level, the glycolysis/gluconeogenesis, and citrate cycle pathways in *P. wettsteinii* leaves were repressed under high-temperature stress since most of the structural genes involved in the pathways were significantly downregulated in SH/CK, whereas *gpmI*, *ALDO*, *tpiA*, *pfkA*, and *ACO* were significantly upregulated in SH/CK. These results might suggest that glycolysis/gluconeogenesis and the citrate cycle pathway were triggered as defence mechanisms in response to high-temperature stress in *P. wettsteinii*.

### Analysis of DEGs Associated With the Antioxidant System

In POD-catalyzed reactions, four *E1.11.1.7*s were all significantly downregulated in SH/CK, one *E1.11.1.7* was significantly downregulated in MH/CK and SH/CK and three *E1.11.1.7*s were all significantly upregulated in MH/CK. In addition, in CAT-catalyzed reactions, two *CAT*s were all significantly downregulated in SH/CK (Figure 5; Supplementary Table S8).

### High-Temperature Damage Induced Secondary Metabolite Accumulation

Principal component analysis was used to analyze the dynamic changes in *P. wettsteinii* under temperature stress. As shown in Supplementary Figure S4, some plots representing samples of *P. wettsteinii* under the CK, LH, MH, and SH treatments clustered together. Other samples showed distinct separation, suggesting significant changes in metabolites in those samples.

**Figure 6:**
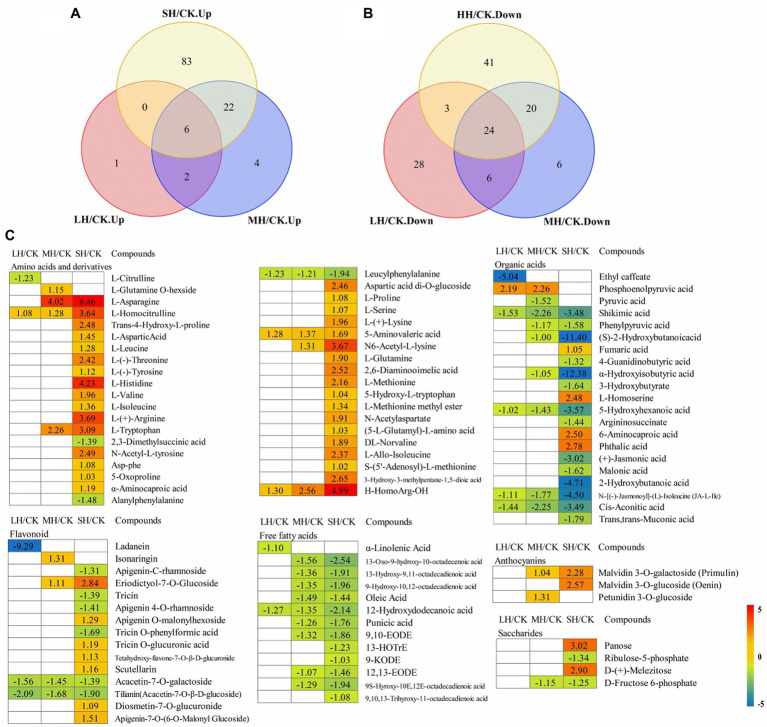
Global view of the distinct and common metabolite target *Paspalum wettsteinii* in response to high-temperature stress. Venn diagram shows metabolites up- **(A)** and downregulated **(B)** by complementary mass spectrometry (GC–MS) analyses in the *P. wettsteinii* leaves. Differentially accumulated metabolites showed expression changes during temperature treatments” with “Heatmap **(C)** shows in expression changes of differentially accumulated metabolites under different temperature treatments. The numbers in the rectangles indicate the log_2_(fold-change) of the comparisons.

To determine the metabolites that differentially accumulated in response to high-temperature stress, nonobjective metabolic spectral analysis was carried out *via* LC–MS. Metabolites with a variable importance in projection (VIP) value > 1 and a value of *p* < 0.05 were considered significantly differentially accumulated metabolites. In total, 246 differentially accumulated metabolites were identified according to a Venn diagram (Figures 6A,B). With increasing temperature, *P. wettsteinii* showed an increased number of metabolites. For LH/CK, 70 metabolites significantly differed (nine upregulated and 61 downregulated); for MH/CK, 90 metabolites significantly differed (34 upregulated and 56 downregulated); and for SH/CK, 199 metabolites significantly differed (111 upregulated and 88 downregulated). Among these differentially accumulated metabolites, 30 were common in all three comparison groups (LH/CK, MH/CK, and SH/CK).

These differential metabolites belonged to amino acids, amino acid derivatives, carbohydrates, anthocyanin, flavonoids, lipid fatty acids, lipid-glycerophospholipids, phytohormones, and organic acids (Supplementary Table S9).

A total of 39 differentially accumulated metabolites involved in amino acid and derivative metabolism were successfully identified under LH, MH, and SH stresses, and most amino acid and derivative contents were significantly increased in SH/CK, with the exception of L-glutamine O-hexside, L-citrulline, 2,3-dimethylsuccinic acid, alanylphenylalanine, and leucylphenylalanine. The L-glutamine O-hexside content was significantly increased in the MH/CK comparison, the contents of 2,3-dimethylsuccinic acid and alanylphenylalanine were significantly decreased in the SH/CK comparison and the leucylphenylalanine content was significantly decreased in the three comparison groups (Figure 6C).

For flavonoids, a total of 15 metabolites presented significant discrepancies in the LH/CK, MH/CK, and SH/CK comparisons. The levels of apigenin-C-rhamnoside, tricin, apigenin 4-O-rhamnoside, and tricin O-phenylformic acid were significantly decreased in the SH/CK comparison, the levels of acacetin-7-O-galactoside and tilianin (acacetin-7-O-β-D-glucoside) were significantly decreased in the three comparison groups, the ladanein level was significantly decreased in LH/CK, the levels of eriodictyol-7-O-glucoside, apigenin O-malonylhexoside, tricin O-glucuronic acid, tetahydroxy-flavone-7-O-β-D-glucuronide, scutellarin, diosmetin-7-O-glucuronide, and apigenin-7-O-(6-O-malonyl glucoside) were significantly increased in SH/CK and the sonaringin level was significantly increased in the MH/CK comparison (Figure 6C).

For organic acids, a total of 21 metabolites were also changed in response to high-temperature stresses. Most organic acid contents were significantly decreased in the SH/CK comparison, with the exception of ethyl caffeate, phosphoenolpyruvic acid, fumaric acid, L-homoserine, 6-aminocaproic acid, and phthalic acid. The ethyl caffeate content was significantly decreased in the LH/CK comparison, the phosphoenolpyruvic acid content was significantly increased in the LH/CK and MH/CK comparisons and fumaric acid, L-homoserine, 6-aminocaproic acid, and phthalic acid were significantly increased in the SH/CK comparison (Figure 6C).

For free fatty acids, a total of 13 metabolites presented significant discrepancies in the LH/CK, MH/CK, and SH/CK comparisons. The levels of most of these metabolites were decreased in the MH/CK and SH/CK comparisons, while α-linolenic acid was significantly decreased in the LH/CK comparison, and 13-HOTrE, 9-KODE, and 9,10,13-trihyroxy-11-octadecadienoic acid were significantly decreased in the SH/CK comparison (Figure 6C).

Four saccharides, panose and D-(+)-melezitose, were significantly increased in the SH/CK comparison, ribulose-5-phosphate was significantly decreased in SH/CK and D-fructose 6-phosphate was significantly decreased in the MH/CK and SH/CK comparisons (Figure 6C).

Three anthocyanins were significantly increased including malvidin 3-O-galactoside (primulin) significantly increased in the MH/CK and SH/CK comparisons, malvidin 3-O-glucoside (oenin) significantly increased in SH/CK and petunidin 3-O-glucoside significantly increased in MH/CK (Figure 6C).

## Discussion

Under high-temperature stress, plants often initiate complex networks of regulatory mechanisms to alter gene expression levels and metabolite accumulation, facilitating the interaction of biochemical and molecular processes within the organism to adapt to the stressful environment (Wu et al., 2009). By systematically investigating the physiological, transcriptional, and metabolic responses to high-temperature stress, we revealed different patterns of response to heat stress in *P. wettsteinii*, including HSPs and transcription factors, biosynthesis of secondary metabolites, antioxidant systems, energy metabolism, and carbohydrate metabolism.

Plants are involved in cellular defence against heat damage mainly through the regulation of gene expression and accumulation of HSPs under heat stress (de Los Reyes and Casas-Tintó, 2022). HSPs, including HSP90 and HSP100, are a class of stress proteins newly synthesized or increased in content by cells or organisms in response to environmental stress and have a key role in regulating protein quality, which in turn can be rapidly activated or repressed under heat stress by being rapidly activated or repressed by TFs such as heat shock factor (HSFs), zinc finger, and WRKYs under heat stress (Farhangi-Abriz and Torabian, 2017; Wang et al., 2022). As the upstream regulatory proteins of HSPs and other molecular chaperones, HSFs play an important regulatory role in the perception and signaling of heat stress (von Koskull-Döring et al., 2007). In this study, transcriptome analysis by RNA-seq detected 28 TF families, including HSFs, zinc finger, and WRKYs, in response to heat stress. Two of these HSFs were upregulated at 45°C. In *Arabidopsis* (Scharf et al., 2018) and tomato (Xu et al., 2013), 21 and 15 HSFs were found to play a central role in the heat stress response, respectively. Overexpression of *HsfA1*, a major member of *Arabidopsis*, soybean, rice, and tomato Hsfs, significantly enhanced their heat tolerance (Grover et al., 2013). Thus, our study suggests that HSF plays an important role in *P. wettsteinii* in response to heat stress. Similarly, our study identified 17 HSPs, including *HSP20*, *HSP90A*, *HSP90B*, *HSPA1*s, *HSPA4*, and *HSPA5*, most of which were significantly upregulated at 45°C. This showed that more HSPs were synthesized in *P. wettsteinii* with the intensification of thermal excitation, which exhibited excellent heat resistance. This also verifies the importance of HSPs in the regulation of thermal excitation.

WRKYs are involved in the temperature stress response by different regulatory mechanisms, both directly binding and regulating the expression of target genes and they also play the role of negative regulators of adversity (Giacomelli et al., 2012; Yuan et al., 2018; Shen et al., 2019). In this study, one WRKY gene (*WRKY72*) was found to be upregulated in *P. wettsteinii*, which may positively regulate heat stress in *P. wettsteinii*; all 15 WRKY genes were downregulated in *P. wettsteinii*, which may be related to the negative regulatory mechanism specific to *P. wettsteinii* or may also interact with other transcription factors to indirectly regulate heat stress. In addition, zinc fingers play an important role in plant stress (Ohama et al., 2017; Hu et al., 2019). In this study, four types of zinc finger proteins, C-x8-C-x5-C-x3-H type, GATA type, Dof domain, and C2H2 type, were identified, and only one C2H2 type gene was downregulated, while the expression levels of most of the genes encoding zinc finger proteins were upregulated. The results of real-time quantitative PCR experiments in tomato showed that the expression patterns of different C2H2 genes were inconsistent under heat stress, with some upregulated and some downregulated (Easterling et al., 2000). These results suggest that heat stress may induce different levels of differential expression of zinc finger in response to heat stress.

More studies have shown that high-temperature stress disrupts the balance between ROS production and scavenging, induces its excessive accumulation and decreases the stability of the plasma membrane (Jendricko et al., 2009; Cui et al., 2013). The changes in membrane lipid components (fatty acids) also reflect the response of plants to environmental stresses (Hill et al., 2013; Xia et al., 2020). In this study, we found that with the enhancement of high-temperature stress, the MDA content first decreased and then increased, and at 35°C, the MDA content was lower than that of CK. It was tentatively speculated that this might be due to the scavenging of excess reactive oxygen radicals produced in *P. wettsteinii* at 35°C and the balance of reactive oxygen metabolism, which was an adaptive response of *P. wettsteinii* manifested at this temperature. In contrast, the MDA content was higher than that of CK under 40 and 45°C high-temperature stress, which is consistent with our metabolomic analysis finding that the content of 13 free fatty acid metabolites was significantly reduced under high-temperature stress and that the number of fatty acids increased with the intensification of high-temperature stress. This indicates that high temperature exacerbates membrane lipid peroxidation and damages the cell membrane. To protect cells from the deleterious effects of excessive ROS, plants have evolved a complex array of enzymatic and non-enzymatic antioxidant defence mechanisms (Muhlemann et al., 2018). SOD, POD, and CAT are important antioxidant enzymes in plants, and their activities are enhanced to scavenge accumulated O^2−^ and thus prevent cell membrane damage (Rugienius et al., 2016). In this study, we found that CAT, SOD, and POD activities increased and then decreased with increasing high-temperature stress, and all of them were significantly lower than CK at 45°C, while POD was higher than CK at 40°C. This is consistent with our finding that the expression of two CAT-related genes and five POD-related genes decreased at 45°C and three POD-related genes (*E1.11.1.7*) increased at 40°C by RNA-seq sequencing. This indicates that the heat tolerance of *P. wettsteinii* was exceeded at 40°C, the balance of ROS metabolism in the body was disrupted and the reactive oxygen species caused damage to the protective enzymes, resulting in the reduction of protective enzyme activity and reactive oxygen species injury.

Plants produce a large number of secondary metabolites under high-temperature stress, most of which are antioxidants, among which flavonoids and anthocyanins are members of the plant’s own formation of non-enzymatic antioxidant defence system and play an important role in the process of resisting adversity (Sperdouli and Moustakas, 2012; Janka et al., 2015; Hao, 2017). In our study, 13 genes involved in the flavonoid biosynthesis pathway encoding catalase genes in flavonoid biosynthesis were found to be differentially expressed, of which two genes encoding 3′-monooxygenases (*CYP75B1* and *CYP75B2*) were upregulated at 35 and 40°C. The remaining 11 genes were all downregulated under high-temperature stress, and the 11 genes included one gene for the synthesis and reduction of bifunctional dihydroflavonol 4-reductase/flavanone 4-reductase (*DFR*), a gene for anthocyanidin synthase (*ANS*), and two genes for anthocyanidin reductase (*ANR*). Inconsistent with the findings of Song et al. (2014), peony subjected to high-temperature stress showed an increase in the expression of secondary metabolites such as flavonoids, isoflavonoids, and anthocyanidins DEGs. This may be due to the decrease in the rate of flavonoid and anthocyanin synthesis in *P. wettsteinii* with increasing temperature and the consequent decrease in the ability of *P. wettsteinii* to scavenge reactive oxygen species and regulate osmotic potential. In the metabolome, 15 flavonoid metabolites were identified, including ladanein, apigenin-C-rhamnoside, tricin, apigenin 4-O-rhamnoside, tricin O-phenylformic acid, acacetin-7-O-galactoside, and tilianin (acacetin-7-O-β-D-glucoside). The accumulation of seven species decreased under high-temperature stress, similar to the results of RNA-seq sequencing, indicating that the antioxidant effect was reduced and the degree of broad membrane lipid peroxidation was severe in broad-leaved barnyardgrass under high-temperature stress. Three anthocyanin metabolites, malvidin 3-O-galactoside (primulin), malvidin 3-O-glucoside (oenin), and petunidin 3-O-glucoside, were found to be upregulated at 40 and 45°C in the metabolome, contrary to the results of RNA-seq sequencing. Shen et al. (2019) found that decreased expression levels of genes involved in the anthocyanin pathway may not lead to reduced anthocyanin accumulation, and further analysis is needed to determine the exact cause.

Photosynthesis is one of the most temperature-sensitive physiological processes in plants. High-temperature stress affects the light capture system, electron transport, NADPH and ATP synthesis, the photosynthetic carbon cycle, and the utilization of assimilates (Liu et al., 2011; Wujeska-Klause et al., 2015). PS II is the most susceptible part of the photosynthetic organ to high temperatures (Alboresi et al., 2008). Under high-temperature stress, several protein subunits in the light and electron transport chain (ETC; e.g., *Psb27* and *Psb28*, etc.) and auxin (e.g., *CP43*) mediate PS II repair (Ifuku et al., 2005). Oxygen evolution complex (OEC) proteins are membrane-exogenous intraluminal subunits of PS II and are involved in the normal function of photosynthetic water oxidation (Arakaki et al., 1997). Expression of the gene encoding photosystem II oxygen evolution enhancer protein 3 (*PsbQ*) was upregulated under treatment at 40 and 45°C, indicating enhanced photosynthetic water oxidation at this temperature. Ferricoxigenin-NADP+ reductase is primarily responsible for catalyzing the final step of the photosynthetic linear electron transfer reaction, catalyzing the transfer of electrons from the reduced state of ferricoxigenin to NADP+ to generate the reducing power ADPH used primarily for Calvin cycle CO_2_ fixation (Oquist and Huner, 2003; Lu et al., 2018). Under 45°C treatment, although the expression of the gene encoding ferredoxin (*petF*) was upregulated, the expression of the ferredoxin--NADP+ reductase gene (*petH*), which is responsible for catalyzing the transfer of ferric oxide-reducing proteins to NADP+ to generate reductive ADPH, was downregulated. This result indicates that genes associated with the light and ETC are inactivated under high-temperature stress, the PS II system is compromised and photosynthesis is inhibited.

Light capture proteins (LHCs) are essential for light capture and photoprotection. The *Lhcal-6* and *Lhcbl-8* LHC subfamilies are associated with PS I and PS II photosynthetic protection, respectively (Carrillo and Ceccarelli, 2003). In this study, six LHCB genes (*LHCB1*, *LHCB2*, *LHCB3*, and *LHCB6*) were downregulated in response to high-temperature stress at 45°C, consistent with an increase and then a decrease in chlorophyll *a* and chlorophyll *b* content with increasing levels of high-temperature stress. This result may be due to the occurrence of photoinhibition under stress conditions, further reducing chlorophyll content and the amount of photosynthetic system antenna proteins, while nonphotochemical quenching was able to reduce the deleterious effects caused by reactive oxygen species (Jennings, 2015). High-temperature stress affects photosynthesis not only through photosystems and LHC protein complexes but also through carbon fixation that inhibits the activity of various biological enzymes (Bibi et al., 2008; Gao et al., 2017). Fructose-1,6-bisphosphatase is a rate-limiting enzyme in the glucose xenobiotic pathway, the level of which directly affects assimilate accumulation and thus plant photosynthetic efficiency (Zhou et al., 2010). At 45°C, the expression of the gene encoding fructose-1,6-bisphosphatase (FBP) is downregulated, indicating a reduction in plant photosynthetic efficiency. Phosphoenolpyruvate carboxylase, pyruvate phosphate dikinase and malate dehydrogenase are all key enzymes in the photosynthetic pathway of C4 plants (Downs and Heckathorn, 1998). The results of this study showed that the gene expression of *ppc*, *ppdK*, and *E1.1.1.82* did not change at 35 and 40°C and was downregulated at 45°C. This result indicated that photosynthetic carbon fixation was not affected at 35 and 40°C, and photosynthetic physiological activity was normal. The differential expression of genes related to photosynthetic processes under different temperature treatments is an adaptation strategy for the tolerance of *P. wettsteinii* to high temperature.

Respiration includes glycolysis/gluconeogenesis, the TCA cycle and the mitochondrial ETC. These pathways can provide the energy equivalent and carbon skeleton for metabolite biosynthesis under high-temperature conditions (Bolton, 2009; Araújo et al., 2012; Yang et al., 2015). In this study, 17 key genes for glycolysis/gluconeogenesis and the TCA cycle were detected by differential expression. Among these genes, the expression of *gpmI*, *ALDO*, *tpiA*, and *PFP*, genes related to the glycolysis/gluconeogenesis pathway, was upregulated, and the succinate dehydrogenase gene *SDHB*, related to the TCA cycle, was downregulated. This indicates that under high-temperature stress, the energy production of *P. wettsteinii* is more variable, with increased glucose consumption as well as reduced carbohydrates, and a shift from photosynthetic phosphorylation to oxidative phosphorylation is the main method of providing ATP (Huang et al., 2019). At the same time, some genes related to glycolysis/gluconeogenesis, *FBP*, *E5.1.3.15*, *ENO*, *GALM*, *E1.1.1.1*, *ALDH*, and *PFP* expression were downregulated, and the aconitate hydratase gene *ACO*, which is related to the TCA cycle, was upregulated under high-temperature stress, indicating that high-temperature stress inhibited these two pathways while enhancing electron transfer and that *P. wettsteinii* plants were able to produce energy through ATP supply was maintained by constantly altering the transcript levels of key enzyme genes in the glycolysis/gluconeogenesis pathway.

Soluble carbohydrates and amino acids are primary metabolites synthesized from intermediate metabolites of glycolysis/gluconeogenesis and the TCA cycle and play an important role in heat stress (Wang et al., 2020). Soluble sugars maintain photosynthetic capacity by maintaining cell membrane stability and keeping a plant viable on their own, protecting the photosynthetic system from damage (Michaletti et al., 2018). Amino acids and their derivatives play an important role in maintaining osmoregulation and the structural integrity of proteins within the cell (Good and Zaplachinski, 2010). Our metabolomic analysis identified 39 amino acids and their derivatives and four sugars. Among the amino acids, 35 (proline, leucine, etc.) significantly increased at high temperatures, and four (L-citrulline, 2,3-dimethylsuccinic acid, alanylphenylalanine, and leucylphenylalanine) significantly decreased under high-temperature stress; among the sugars, the contents of panose and D-(+)-melezitose significantly increased, and those of ribulose-5-phosphate and D-fructose 6-phosphate significantly decreased. This suggests that in glycolysis/gluconeogenesis and the TCA cycle, high temperatures can increase sucrose and pyruvate levels, while also decreasing the levels of certain phosphate sugars and organic acids to maintain osmotic balance (Yamakawa and Hakata, 2010; Hu et al., 2014). Organic acids were found to be involved in glycolysis and the TCA cycle, and we identified 18 organic acids by metabolomic analysis, most of which were significantly reduced at 40 and 45°C. In contrast, Yamakawa and Hakata (2010) found that high-temperature stress reduced the content of organic acids involved in glycolysis/gluconeogenesis and the tricarboxylic acid cycle in rice seeds, contrary to our results, which may be because *P. wettsteinii* is a warm-season plant and *P. wettsteinii* adapts to high-temperature stress by increasing the organic acid content.

**Figure 7:**
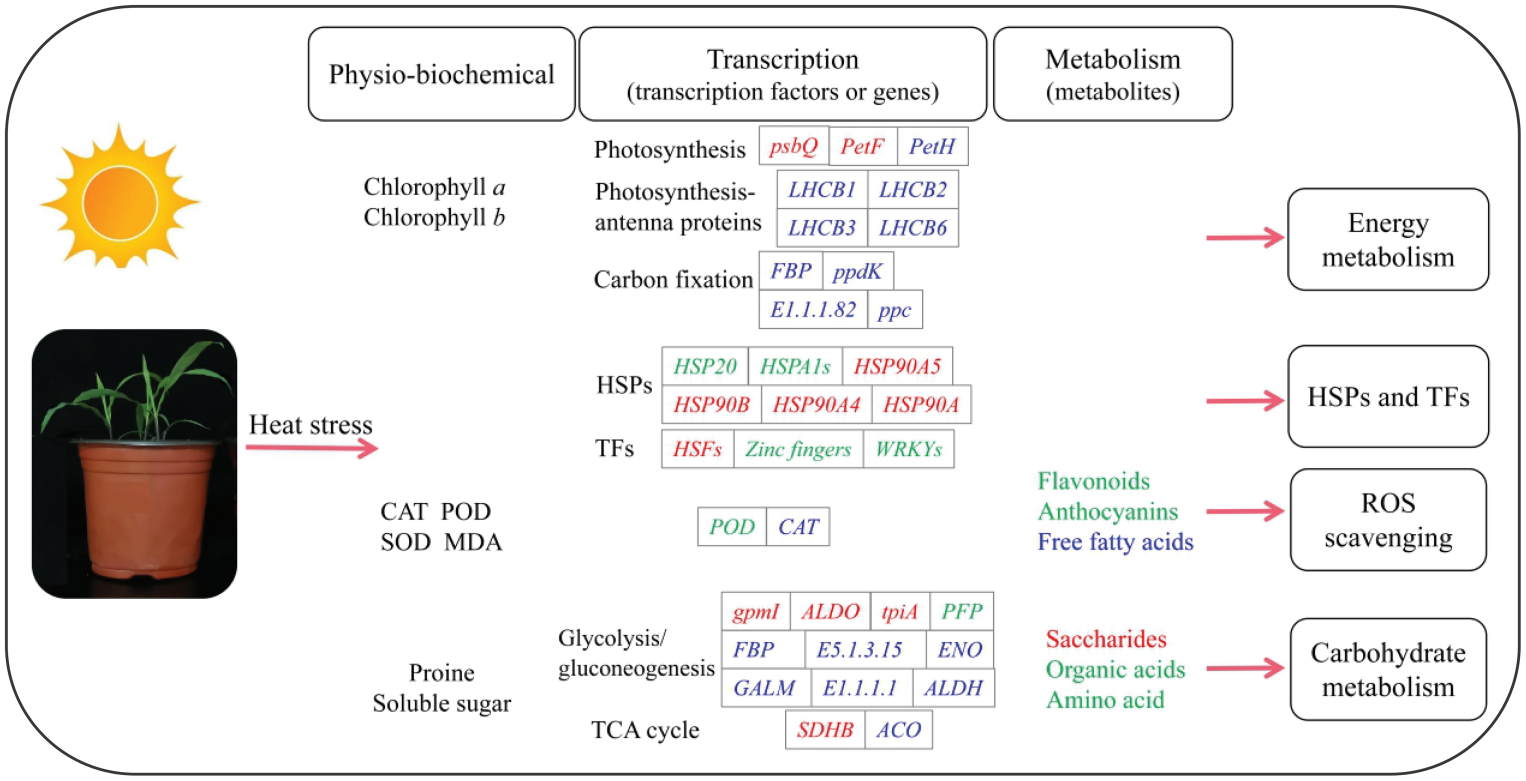
A schematic diagram summarizing the responses to high-temperature stress in *Paspalum wettsteinii*. The changes of main indexes in Physio-biochemical, transcription, and metabolism are listed. The genes and metabolites in red, blue, or green letters refer to up-, down- and both up- and downregulated expression, respectively, in response to high temperature.

Our study provides detailed information on the physio-biochemical, transcription and metabolism of *P. wettsteinii* in response to high-temperature stress (Figure 7). Based on these multilevel results, we conclude that, on the one hand, *P. wettsteinii* slowed down the plant height growth, which showed a decrease in antioxidant enzyme activity and non-enzymatic antioxidant substances accumulation, which weakened the ability of antioxidants to scavenge ROS in *P. wettsteinii*. On the other hand, *P. wettsteinii* responded to high-temperature stress by suppressing some genes related to the light capture complex and electron transport system in photosynthesis, enhancing the glycolytic pathway in respiration, inhibiting the TCA cycle, and inducing transcription factors to regulate HSPs.

## Conclusion

To investigate the physiological and biochemical characteristics, key candidate genes and metabolites of *P. wettsteinii* under high-temperature stress were identified. Plant height, CAT, SOD, and POD activities, and SS, PS, chlorophyll *a*, and chlorophyll *b* contents tended to increase and then decrease, while the MDA content tended to decrease and then increase with increasing heat stress. In addition, a multilevel analysis of gene/metabolite expression changes in *P. wettsteinii* was carried out. Genes involved in important energy metabolism, carbohydrate metabolism, heat shock protein, and transcription factors, biosynthesis of secondary metabolites and antioxidant system were significantly up- and downregulated to varying degrees. Free fatty acids, amino acids, and their derivatives, organic acids, flavonoids, and sugars were found to accumulate to varying degrees under heat stress. The integration of transcriptomic and metabolomic data will provide theoretical support for further studies on the complex mechanisms underlying the response to high-temperature stress in *P. wettsteinii* and other plants.

## Data Availability Statement

The datasets presented in this study can be found in online repositories. The name of the repository and accession number can be found at: SRA, NCBI; PRJNA801753.

## Author Contributions

XZ: experimental design, experimental performance, experimental data collection, data analysis, and manuscript writing and revision. L-LZ: seed provision, manuscript writing, resource provision, and funding acquisition. L-JH and X-FS: experimental performance, experimental data collection, and data analysis. P-CW: manuscript writing and revision. All authors contributed to the article and approved the submitted version.

## Funding

This work was funded through projects of Science and Technology Project of Guizhou Province (QKHJC[2020]1Z026, QKHZC[2021]YB155, QKHPTRC[2021]5636), and the National Natural Science Foundation of China (32060391 and 31702173).

## Conflict of Interest

The authors declare that the research was conducted in the absence of any commercial or financial relationships that could be construed as a potential conflict of interest.

## Publisher’s Note

All claims expressed in this article are solely those of the authors and do not necessarily represent those of their affiliated organizations, or those of the publisher, the editors and the reviewers. Any product that may be evaluated in this article, or claim that may be made by its manufacturer, is not guaranteed or endorsed by the publisher.

## Footnotes

^1^ http://www.geneontology.org/

^2^ http://www.genome.jp/kegg/

^3^ https://www.genome.jp/kegg/pathway.html

## References

[ref1] Alboresi A. Caffarri S. Nogue F. Bassi R. Morosinotto T. (2008). In silico and biochemical analysis of *Physcomitrella patens* photosynthetic antenna: identification of subunits which evolved upon land adaptation. PLoS One 3:e2033. doi: 10.1371/journal.pone.0002033, PMID: 18446222 PMC2323573

[ref2] Almeselmani M. Deshmukh P. S. Sairam R. K. (2015). High temperature stress tolerance in wheat genotypes: role of antioxidant defence enzymes. Acta Agron. Hung. 57, 1–14. doi: 10.1556/AAgr.57.2009.1.1

[ref3] Arakaki A. K. Ceccarelli E. A. Carrillo N. (1997). Plant-type ferredoxin-NADP+ reductases: a basal structural framework and a multiplicity of functions. FASEB J. 11, 133–140. doi: 10.1096/fasebj.11.2.9039955, PMID: 9039955

[ref4] Aranguren-Gassis M. Kremer C. T. Klausmeier C. A. Litchman E. (2019). Nitrogen limitation inhibits marine diatom adaptation to high temperatures. Ecol. Lett. 22, 1860–1869. doi: 10.1111/ele.13378, PMID: 31429516

[ref5] Araújo W. L. Nunes-Nesi A. Nikoloski Z. Sweetlove L. J. Fernie A. R. (2012). Metabolic control and regulation of the tricarboxylic acid cycle in photosynthetic and heterotrophic plant tissues. Plant Cell Environ. 35, 1–21. doi: 10.1111/j.1365-3040.2011.02332.x, PMID: 21477125

[ref6] Bañon S. Fernandez J. A. Franco J. A. Torrecillas A. Alarcón J. J. Sánchez-Blanco M. J. (2004). Effects of water stress and night temperature preconditioning on water relations and morphological and anatomical changes of lotus creticus plants. Sci. Hortic. 101, 333–342. doi: 10.1016/j.scienta.2003.11.007

[ref7] Bibi A. C. Oosterhuis D. M. Gonias E. D. (2008). Photosynthesis, quantum yield of photosystem ii and membrane leakage as affected by high temperatures in cotton genotypes. J. Cotton Sci. 12, 150–159.

[ref8] Bolton M. D. (2009). Primary metabolism and plant defense-fuel for the fire. Mol. Plant-Microbe Interact. 22, 487–497. doi: 10.1094/MPMI-22-5-0487, PMID: 19348567

[ref9] Carrillo N. Ceccarelli E. A. (2003). Open questions in ferredoxin-NADP+ reductase catalytic mechanism. Eur. J. Biochem. 270, 1900–1915. doi: 10.1046/j.1432-1033.2003.03566.x, PMID: 12709048

[ref10] Chang J. Shi J. Lin J. Ji D. Xie C. (2021). Molecular mechanism underlying pyropia haitanensis phhsp22-mediated increase in the high-temperature tolerance of *Chlamydomonas reinhardtii*. J. Appl. Phycol. 33, 1137–1148. doi: 10.1007/s10811-020-02351-6

[ref11] Chebrolu K. K. Fritschi F. B. Ye S. Krishnan H. B. Smith J. R. Gillman J. D. (2016). Impact of heat stress during seed development on soybean seed metabolome. Metabolomics 12:28. doi: 10.1007/s11306-015-0941-1

[ref12] Chen C. Li P. Wang W. Li Z. (2022). Response of growth performance, serum biochemical parameters, antioxidant capacity, and digestive enzyme activity to different feeding strategies in common carp (*Cyprinus carpio*) under high-temperature stress. Aquaculture 548:737636. doi: 10.1016/j.aquaculture.2021.737636

[ref13] Cui W. Ma J. Wang X. Yang W. Zhang J. Ji Q. (2013). Free fatty acid induces endoplasmic reticulum stress and apoptosis of β-cells by Ca^2+^/calpain-2 pathways. PLoS One 8:e59921. doi: 10.1371/journal.pone.0059921, PMID: 23527285 PMC3604010

[ref14] de Los Reyes T. Casas-Tintó S. (2022). Neural functions of small heat shock proteins. Neural Regen. Res. 17, 512–515. doi: 10.4103/1673-5374.320975, PMID: 34380880 PMC8504394

[ref15] Downs C. A. Heckathorn S. A. (1998). The mitochondrial small heat-shock protein protects NADH:ubiquinone oxidoreductase of the electron transport chain during heat stress in plants. FEBS Lett. 430, 246–250. doi: 10.1016/s0014-5793(98)00669-3, PMID: 9688548

[ref16] Du H. Wang Z. Yu W. Liu Y. Huang B. (2011). Differential metabolic responses of perennial grass *Cynodon transvaalensis*×*Cynodon dactylon* (C₄) and *Poa Pratensis* (C₃) to heat stress. Physiol. Plant. 141, 251–264. doi: 10.1111/j.1399-3054.2010.01432.x, PMID: 21114672

[ref17] Easterling D. R. Meehl G. A. Parmesan C. Changnon S. A. Karl T. R. Mearns L. O. (2000). Climate extremes: observations, modeling, and impacts. Science 289, 2068–2074. doi: 10.1126/science.289.5487.2068, PMID: 11000103

[ref18] Escandón M. Meijón M. Valledor L. Pascual J. Pinto G. Cañal M. J. (2018). Metabolome integrated analysis of high-temperature response in *Pinus radiata*. Front. Plant Sci. 9:485. doi: 10.3389/fpls.2018.00485, PMID: 29719546 PMC5914196

[ref19] Farhangi-Abriz S. Torabian S. (2017). Antioxidant enzyme and osmotic adjustment changes in bean seedlings as affected by biochar under salt stress. Ecotoxicol. Environ. Saf. 137, 64–70. doi: 10.1016/j.ecoenv.2016.11.029, PMID: 27915144

[ref20] Gao G. Hu J. Zhang X. Zhang F. Wu X. (2021). Transcriptome analyses reveals genes expression pattern of seed response to heat stress in *Brassica napus* L. Oil Crop Sci. 6, 87–96. doi: 10.1016/j.ocsci.2021.04.005

[ref21] Gao Y. Jiang Z. Du M. Fang J. Jiang W. Fang J. (2019). Photosynthetic and metabolic responses of eelgrass *Zostera marina* L. to short-term high-temperature exposure. J. Ocean. Limnol. 37, 199–209. doi: 10.1007/s00343-019-7319-6

[ref22] Gao G. Jin P. Liu N. Li F. Tong S. Hutchins D. A.. (2017). The acclimation process of phytoplankton biomass, carbon fixation and respiration to the combined effects of elevated temperature and pCO_2_ in the northern South China Sea. Mar. Pollut. Bull. 118, 213–220. doi: 10.1016/j.marpolbul.2017.02.063, PMID: 28259422

[ref23] Giacomelli J. I. Weigel D. Chan R. L. Manavella P. A. (2012). Role of recently evolved miRNA regulation of sunflower *HaWRKY6* in response to temperature damage. New Phytol. 195, 766–773. doi: 10.1111/j.1469-8137.2012.04259.x, PMID: 22846054

[ref24] Good A. G. Zaplachinski S. T. (2010). The effects of drought stress on free amino acid accumulation and protein synthesis in brassica napus. Physiol. Plant. 90, 9–14. doi: 10.1111/j.1399-3054.1994.tb02185.x

[ref25] Grover A. Mittal D. Negi M. Lavania D. (2013). Generating high temperature tolerant transgenic plants: achievements and challenges. Plant Sci. 205–206, 38–47. doi: 10.1016/j.plantsci.2013.01.005, PMID: 23498861

[ref26] Hao Z. (2017). Response to High Temperature Stress and its Chemicial Regulation Mechanisms in Herbaceous Peony (*Paeonia lactiflora* Pall.). Yangzhou University.

[ref27] Herman D. J. Knowles L. O. Knowles N. R. (2017). Heat stress affects carbohydrate metabolism during cold-induced sweetening of potato (*Solanum tuberosum* L.). Planta 245, 563–582. doi: 10.1007/s00425-016-2626-z, PMID: 27904974

[ref28] Hill M. N. Kumar S. A. Filipski S. B. Iverson M. Stuhr K. L. Keith J. M.. (2013). Disruption of fatty acid amide hydrolase activity prevents the effects of chronic stress on anxiety and amygdalar microstructure. Mol. Psychiatry 18, 1125–1135. doi: 10.1038/mp.2012.90, PMID: 22776900 PMC4148304

[ref29] Hu T. Sun X. Zhang X. Nevo E. Fu J. (2014). An RNA sequencing transcriptome analysis of the high-temperature stressed tall fescue reveals novel insights into plant thermotolerance. BMC Genomics 15:1147. doi: 10.1186/1471-2164-15-1147, PMID: 25527327 PMC4378353

[ref30] Hu T. Sun X. Y. Zhao Z. J. Amombo E. Fu J. M. (2020). High temperature damage to fatty acids and carbohydrate metabolism in tall fescue by coupling deep transcriptome and metabolome analysis. Ecotoxicol. Environ. Saf. 203:110943. doi: 10.1016/j.ecoenv.2020.110943, PMID: 32678750

[ref31] Hu X. Zhu L. Zhang Y. Xu L. Li N. Zhang X.. (2019). Genome-wide identification of C2H2 zinc-finger genes and their expression patterns under heat stress in tomato (*Solanum lycopersicum* L.). PeerJ 7:e7929. doi: 10.7717/peerj.7929, PMID: 31788352 PMC6882421

[ref32] Huang M. Zhang H. Zhao C. Chen G. Zou Y. (2019). Amino acid content in rice grains is affected by high temperature during the early grain-filling period. Sci. Rep. 9:2700. doi: 10.1038/s41598-019-38883-2, PMID: 30804353 PMC6389877

[ref33] Ifuku K. Yamamoto Y. Ono T. A. Ishihara S. Sato F. (2005). PsbP protein, but not PsbQ protein, is essential for the regulation and stabilization of photosystem II in higher plants. Plant Physiol. 139, 1175–1184. doi: 10.1104/pp.105.068643, PMID: 16244145 PMC1283756

[ref34] Janka E. Körner O. Rosenqvist E. Ottosen C. O. (2015). Using the quantum yields of photosystem II and the rate of net photosynthesis to monitor high irradiance and temperature stress in chrysanthemum (*Dendranthema grandiflora*). Plant Physiol. Biochem. 90, 14–22. doi: 10.1016/j.plaphy.2015.02.019, PMID: 25749731

[ref35] Jendricko T. Vidović A. Grubisić-Ilić M. Romić Z. Kovacić Z. Kozarić-Kovacić D. (2009). Homocysteine and serum lipids concentration in male war veterans with posttraumatic stress disorder. Prog. Neuro-Psychopharmacol. Biol. Psychiatry 33, 134–140. doi: 10.1016/j.pnpbp.2008.11.002, PMID: 19038303

[ref36] Jennings R. D. (2015). Inorganic Carbon Fixation and Trophic Interactions in High-Temperature Geothermal Springs of Yellowstone National Park, WY, USA. Dissertations and Theses—Gradworks.

[ref37] Kim J. S. Jeon B. W. Kim J. (2021). Signaling peptides regulating abiotic stress responses in plants. Front. Plant Sci. 12:704490. doi: 10.3389/fpls.2021.704490, PMID: 34349774 PMC8326967

[ref38] Liao J. L. Zhou H. W. Peng Q. Zhong P. A. Zhang H. Y. He C.. (2015). Transcriptome changes in rice (*Oryza sativa* L.) in response to high night temperature stress at the early milky stage. BMC Genomics 16:18. doi: 10.1186/s12864-015-1222-0, PMID: 25928563 PMC4369907

[ref39] Liu H. Huang R. Y. Chen J. Gross M. L. Pakrasi H. B. (2011). Psb27, a transiently associated protein, binds to the chlorophyll binding protein CP43 in photosystem II assembly intermediates. Proc. Natl. Acad. Sci. U. S. A. 108, 18536–18541. doi: 10.1073/pnas.1111597108, PMID: 22031695 PMC3215006

[ref40] Lu B. Inoue M. Abe T. (2018). Hydrogen production from methane at 200 to 500°C-clean hydrogen production in conjunction with carbon fixation at 200 to 250°C. Sustain. Energy Fuels 2, 795–802. doi: 10.1039/C7SE00572E

[ref41] Ma Y. Min L. Wang J. Li Y. Wu Y. Hu Q.. (2021). A combination of genome-wide and transcriptome-wide association studies reveals genetic elements leading to male sterility during high temperature stress in cotton. New Phytol. 231, 165–181. doi: 10.1111/nph.17325, PMID: 33665819 PMC8252431

[ref42] Meng L. X. Liang X. Q. Min L. Lu J. X. Li X. N. Li J. B. (2012). Study on preparation of xylan from high-temperature boliled pretreated sugarcane leaves. Food Ferment. Indus. 38, 105–108. doi: 10.13995/j.cnki.11-1802/ts.2012.01.037

[ref43] Michaletti A. Naghavi M. R. Toorchi M. Zolla L. Rinalducci S. (2018). Metabolomics and proteomics reveal drought-stress responses of leaf tissues from spring-wheat. Sci. Rep. 8:5710. doi: 10.1038/s41598-018-24012-y, PMID: 29632386 PMC5890255

[ref44] Muhlemann J. K. Younts T. Muday G. K. (2018). Flavonols control pollen tube growth and integrity by regulating ROS homeostasis during high-temperature stress. Proc. Natl. Acad. Sci. U. S. A. 115, E11188–E11197. doi: 10.1073/pnas.1811492115, PMID: 30413622 PMC6255205

[ref45] Ohama N. Sato H. Shinozaki K. Yamaguchi-Shinozaki K. (2017). Transcriptional regulatory network of plant heat stress response. Trends Plant Sci. 22, 53–65. doi: 10.1016/j.tplants.2016.08.015 27666516

[ref46] Oquist G. Huner N. P. (2003). Photosynthesis of overwintering evergreen plants. Annu. Rev. Plant Biol. 54, 329–355. doi: 10.1146/annurev.arplant.54.072402.115741, PMID: 14502994

[ref47] Rugienius R. Bendokas V. Kazlauskait E. Siksnianas T. Sasnauskas A. (2016). Anthocyanin content in cultivated *Fragaria vesca* berries under high temperature and water deficit stress. Acta Hortic. 1139, 639–644. doi: 10.17660/ActaHortic.2016.1139.110

[ref48] Scharf K. D. Berberich T. Ebersberger I. Nover L. (2018). Corrigendum to "the plant heat stress transcription factor (Hsf) family: structure, function and evolution" [BBAGRM 1819 (2) 104-119]. Biochim. Biophys. Acta Gene Regul. Mech. 1861:60. doi: 10.1016/j.bbagrm.2017.12.005, PMID: 29247802

[ref49] Sezgin M. A. Kadioglu A. (2021). Role of abscisic acid, osmolytes and heat shock factors in high temperature thermotolerance of *Heliotropium thermophilum*. Physiol. Mol. Biol. Plants 27, 861–871. doi: 10.1007/s12298-021-00975-7, PMID: 33967468 PMC8055806

[ref50] Shen J. Zhang D. Zhou L. Zhang X. Liao J. Duan Y.. (2019). Transcriptomic and metabolomic profiling of *Camellia sinensis* L. cv. 'Suchazao' exposed to temperature stresses reveals modification in protein synthesis and photosynthetic and anthocyanin biosynthetic pathways. Tree Physiol. 39, 1583–1599. doi: 10.1093/treephys/tpz059, PMID: 31135909

[ref51] Song Y. Chen Q. Ci D. Shao X. Zhang D. (2014). Effects of high temperature on photosynthesis and related gene expression in poplar. BMC Plant Biol. 14:111. doi: 10.1186/1471-2229-14-111, PMID: 24774695 PMC4036403

[ref52] Sperdouli I. Moustakas M. (2012). Interaction of proline, sugars, and anthocyanins during photosynthetic acclimation of *Arabidopsis thaliana* to drought stress. J. Plant Physiol. 169, 577–585. doi: 10.1016/j.jplph.2011.12.015, PMID: 22305050

[ref53] Sun X. F. Huang L. J. Wang P. C. Zhao L. L. Liu F. (2020). Effects of different phosphorus supply levels on morphology and physiology of *Paspalum wettsteinii*. Acta Pratacul. Sin. 29, 58–69. doi: 10.11686/cyxb2019471

[ref54] von Koskull-Döring P. Scharf K. D. Nover L. (2007). The diversity of plant heat stress transcription factors. Trends Plant Sci. 12, 452–457. doi: 10.1016/j.tplants.2007.08.014, PMID: 17826296

[ref55] Wahid A. Close T. J. (2007). Expression of dehydrins under heat stress and their relationship with water relations of sugarcane leaves. Biol. Plant. 51, 104–109. doi: 10.1007/s10535-007-0021-0

[ref56] Wan L. Lei Y. Yan L. Liu Y. Pandey M. K. Wan X.. (2020). Transcriptome and metabolome reveal redirection of flavonoids in a white testa peanut mutant. BMC Plant Biol. 20:161. doi: 10.1186/s12870-020-02383-7, PMID: 32293272 PMC7161308

[ref57] Wang X. Fang Z. Zhao D. Tao J. (2022). Effects of high-temperature stress on photosynthetic characteristics and antioxidant enzyme system of *Paeonia ostii*. Phyton-Int. J. Exp. Bot. 91, 599–615. doi: 10.32604/phyton.2022.017881

[ref58] Wang L. Ma K. B. Lu Z. G. Ren S. X. Jiang H. R. Cui J. W.. (2020). Differential physiological, transcriptomic and metabolomic responses of *Arabidopsis* leaves under prolonged warming and heat shock. BMC Plant Biol. 20:86. doi: 10.1186/s12870-020-2292-y, PMID: 32087683 PMC7036190

[ref59] Wu X. Shiroto Y. Kishitani S. Ito Y. Toriyama K. (2009). Enhanced heat and drought tolerance in transgenic rice seedlings overexpressing OsWRKY11 under the control of HSP101 promoter. Plant Cell Rep. 28, 21–30. doi: 10.1007/s00299-008-0614-x, PMID: 18818929

[ref60] Wujeska-Klause A. Bossinger G. Tausz M. (2015). Seedlings of two acacia species from contrasting habitats show different photoprotective and antioxidative responses to drought and heatwaves. Ann. Forest Sci. 72, 403–414. doi: 10.1007/s13595-014-0438-5

[ref61] Xia F. Han Z. Zhu H. Dong K. Du L. (2020). Comparison of osmoprotectants and antioxidant enzymes of different wild Kentucky bluegrass in Shanxi province under high-temperature stress. Eur. J. Hortic. Sci. 85, 284–292. doi: 10.17660/eJHS.2020/85.4.10

[ref62] Xu J. Xue C. Xue D. Zhao J. Gai J. Guo N.. (2013). Overexpression of GmHsp90s, a heat shock protein 90 (Hsp90) gene family cloning from soybean, decrease damage of abiotic stresses in *Arabidopsis thaliana*. PLoS One 8:e69810. doi: 10.1371/journal.pone.0069810, PMID: 23936107 PMC3723656

[ref63] Yamada K. Nishimura M. (2008). Cytosolic heat shock protein 90 regulates heat shock transcription factor in *Arabidopsis thaliana*. Plant Signal. Behav. 3, 660–662. doi: 10.4161/psb.3.9.5775, PMID: 19704818 PMC2634549

[ref64] Yamakawa H. Hakata M. (2010). Atlas of rice grain filling-related metabolism under high temperature: joint analysis of metabolome and transcriptome demonstrated inhibition of starch accumulation and induction of amino acid accumulation. Plant Cell Physiol. 51, 795–809. doi: 10.1093/pcp/pcq034, PMID: 20304786 PMC2871029

[ref65] Yang J. Chen X. R. Zhu C. L. Peng X. S. Xiao-Peng H. E. Jun-Ru F. U.. (2015). Effects of nitrogen level and high temperature treatment on yield, spad value, and soluble sugar content of early rice Ganxin 203. Acta Agric. Univ. Jiangxiensis 37, 759–764. doi: 10.13836/j.jjau.2015115

[ref66] Yuan X. Huang P. Wang R. Li H. Lv X. Duan M.. (2018). A zinc finger transcriptional repressor confers pleiotropic effects on rice growth and drought tolerance by Down-regulating stress-responsive genes. Plant Cell Physiol. 59, 2129–2142. doi: 10.1093/pcp/pcy133, PMID: 30020522

[ref67] Zhao J. He Q. Chen G. Wang L. Jin B. (2016). Regulation of non-coding RNAs in heat stress responses of plants. Front. Plant Sci. 7:1213. doi: 10.3389/fpls.2016.01213, PMID: 27588021 PMC4988968

[ref68] Zhao W. Ma Z. Liu S. Yang W. Ma J. (2021). Transcriptome profiling reveals potential genes and pathways supporting *Ananas comosus* L. merr's high temperature stress tolerance. Trop. Plant Biol. 14, 132–142. doi: 10.1007/s12042-021-09287-2

[ref69] Zhao X. Wang W. J. Wang P. C. Huang L. J. Zhao L. L. (2019). Effects of different calcium concentrations on growth and physiology of *Paspalum wettsteinii* seedlings. Chin. J. Plant Ecol. 43, 909–920. doi: 10.17521/cjpe.2019.0235

[ref70] Zhou H. H. Chen Y. N. Li W. H. Chen Y. P. (2010). Photosynthesis of *Populus euphratica* in relation to groundwater depths and high temperature in arid environment, northwest China. Photosynthetica 48, 257–268. doi: 10.1007/s11099-010-0032-5

