# Supplementary material for: Differential Physiological, Transcriptomic, and Metabolomic Responses of Paspalum wettsteinii Under High-Temperature Stress
Source: Front Plant Sci. 2022 Apr 21;13:865608. doi: 10.3389/fpls.2022.865608 (PMC9069066; doi:10.3389/fpls.2022.865608)
Supplement: Supplementary file 1 [file Data_Sheet_1.docx]

**Fig. S1** Scatter plot showing the changes in the expression [log2(Ratio)] of selected genes based on RNA-seq via qRT–PCR.


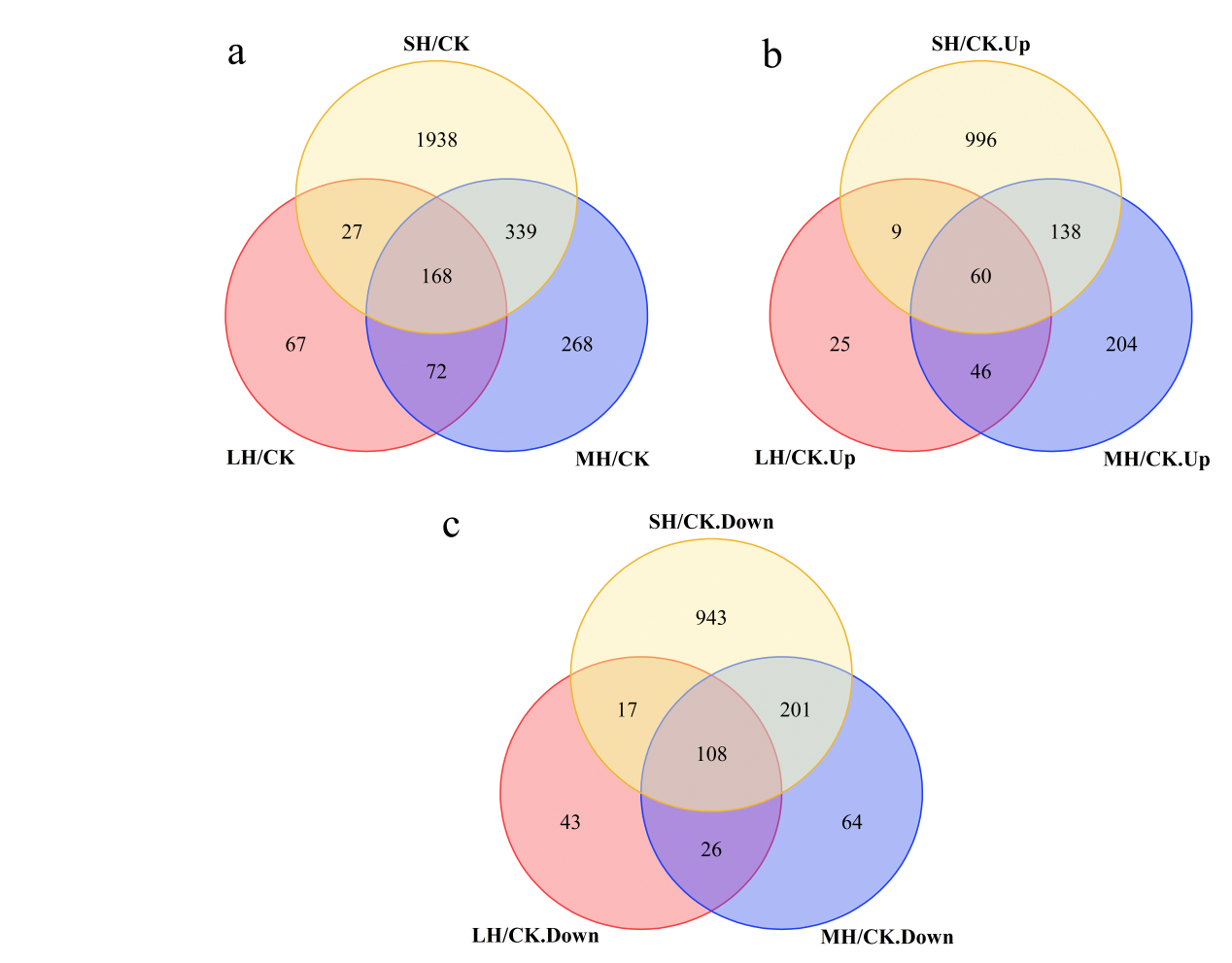


**Fig. S2** DEGs are shown in Venn diagram form. **a** Venn diagram showing the total number of total DEGs, **b** only upregulated DEGs and **c** only downregulated DEGs in *P. wettsteinii* in the LH/CK, MH/CK and SH/CK comparison groups.


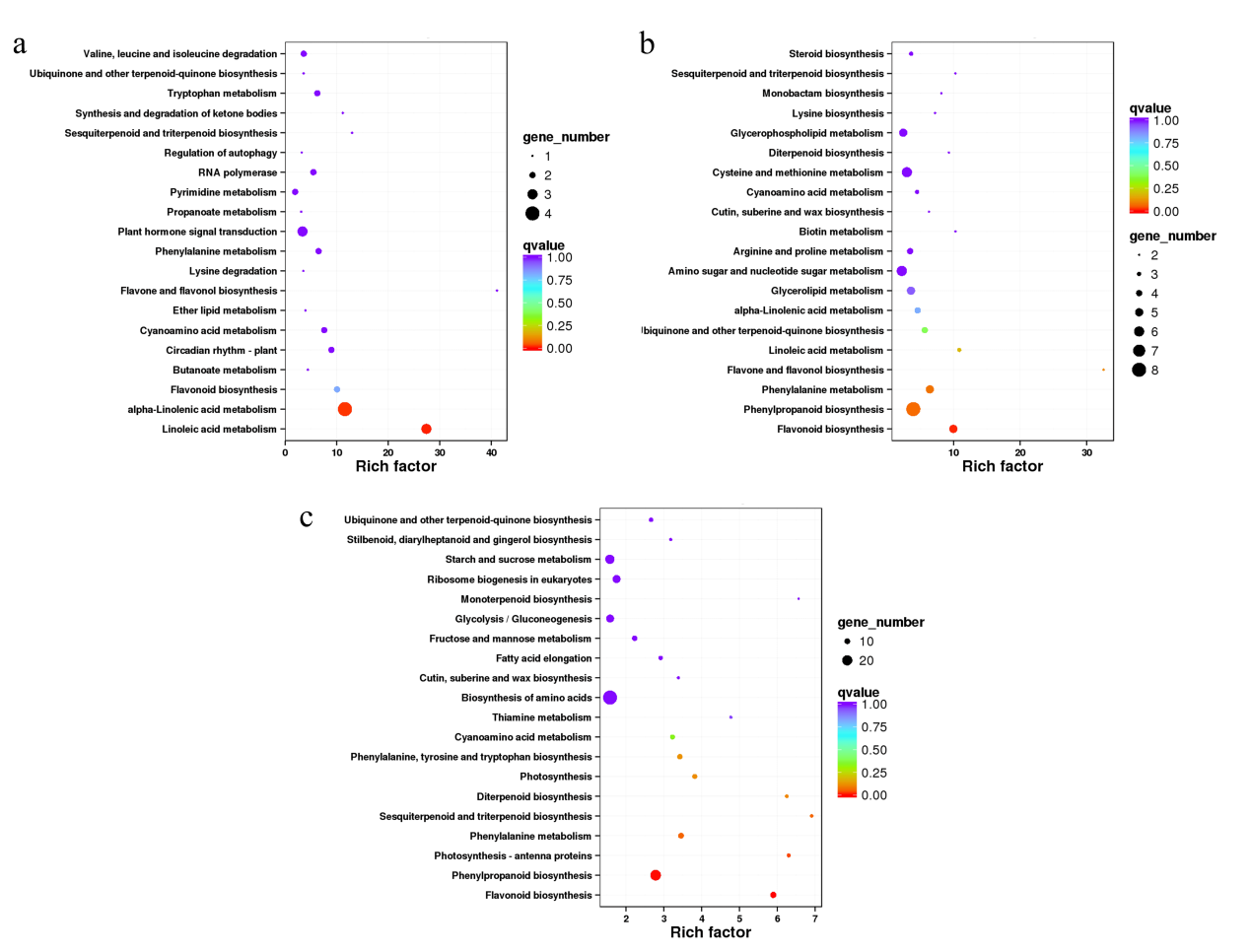


**Fig. S3** KEGG pathway analysis of DEGs. **a** Top 20 enriched KEGG pathways in the LH/CK comparison group. **b** Top 20 enriched KEGG terms in the MH/CK comparison group. **c** Top 20 enriched KEGG terms in the SH/CK comparison group.


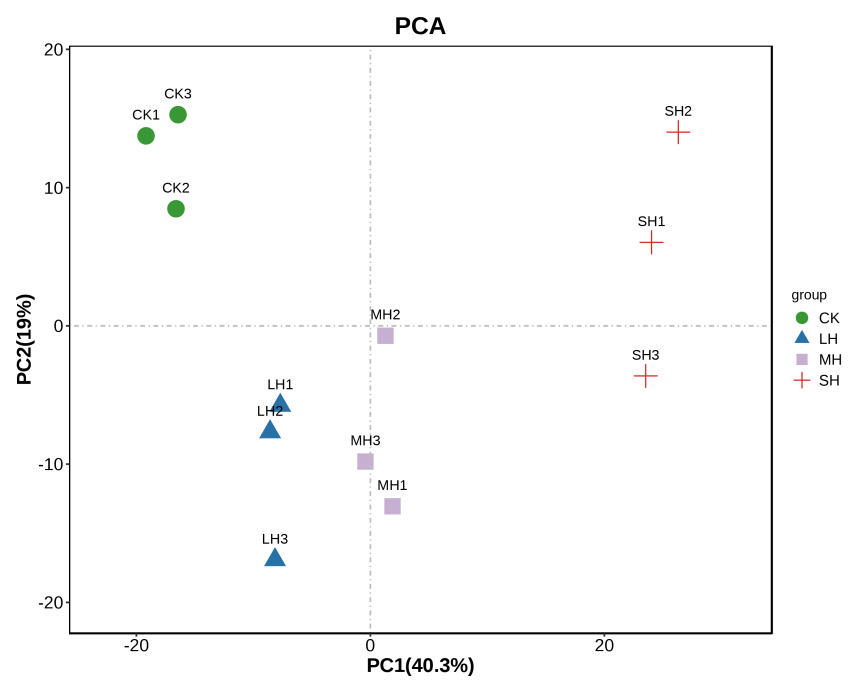


**Fig. S4** Principal component analysis (PCA) of metabolites.
